# Regulatory T Cells Suppress Natural Killer Cells during Plasmid DNA Vaccination in Mice, Blunting the CD8^+^ T Cell Immune Response by the Cytokine TGFβ

**DOI:** 10.1371/journal.pone.0012281

**Published:** 2010-08-19

**Authors:** Kwesi Frimpong-Boateng, Nico van Rooijen, Ralf Geiben-Lynn

**Affiliations:** 1 Division of Viral Pathogenesis, Harvard Medical School, Beth Israel Deaconess Medical Center, Boston, Massachusetts, United States of America; 2 Department of Molecular Cell Biology, Vrije University Medical Center, Amsterdam, The Netherlands; New York University, United States of America

## Abstract

**Background:**

CD4^+^CD25^+^ regulatory T cells (Tregs) suppress adaptive T cell-mediated immune responses to self- and foreign-antigens. Tregs may also suppress early innate immune responses to vaccine antigens and might decrease vaccine efficacy. NK and NKT cells are the first responders after plasmid DNA vaccination and are found at the site of inoculation. Earlier reports demonstrated that NKT cells could improve plasmid DNA efficacy, a phenomenon not found for NK cells. In fact, it has been shown that under certain disease conditions, NK cells are suppressed by Tregs via their release of IL-10 and/or TGFβ. Therefore, we tested the hypothesis that NK cell function is suppressed by Tregs in the setting of plasmid DNA vaccination.

**Methodology/Principal Findings:**

In this study we show that Tregs directly inhibit NK cell function during plasmid DNA vaccination by suppressing the potentially 10-fold, NK cell-mediated, augmentation of plasmid DNA antigen-specific CD8^+^ T cells. We found that this phenomenon is dependent on the secretion of cytokine TGFβ by Tregs, and independent of IL-10.

**Conclusions:**

Our data indicate a crucial function for Tregs in blocking plasmid DNA vaccine-elicited immune responses, revealing potentially novel strategies for improving the efficiency of plasmid DNA vaccines including chemical- or antibody-induced localized blockage of Treg-mediated suppression of NK cells at the site of plasmid DNA vaccine inoculation.

## Introduction

CD4^+^CD25^+^ regulatory T cells (Tregs) represent 5–10% of all CD4^+^ T lymphocytes [1], [2]. They are a unique population of T cells that maintain immune tolerance and are critical to host suppression of autoimmunity [1], [3], [4]. Tregs inhibit the proliferation and effector functions of conventional CD4^+^ and CD8^+^ T lymphocytes [2], [5], [6], natural killer T (NKT) cells [7], B cells [8], dendritic cells (DC) [9], natural killer (NK) cells [10], [11] and cells of the monocyte/macrophage lineage [12].

Tregs play a major role in diseases. During *Helicobacter hepaticus* infection, Tregs elicit tolerance and suppress bacteria-induced colitis [13]. Tregs also control *Leishmania major* persistence and immunity [14]. Recipient-type specific Tregs favor immune reconstitution and control graft-*versus*-host disease without inhibiting graft-*versus*-leukemia immune responses [15]. Additionally, it is thought that Tregs may suppress immune responses against tumors in cancer patients, as increased levels of peripheral and tumor-infiltrating Tregs are predictive of poor survival [16], [17].

Despite their importance, the precise mechanism of Tregs' regulatory activity is still controversial and depends on the *in vitro* or *in vivo* systems investigated. *In vivo*, the inhibitory effects of Tregs are mediated by TGFβ and IL-10 [13], [18]; however, *in vitro*, direct cell-to-cell contact between Tregs and the suppressed cells is necessary. *In vitro*, in contrast to *in vivo*, inhibition is independent of TGFβ or IL-10 [1], .

Tregs might affect the potency of vaccines with respect to both vaccine-induced self-antigen or foreign-antigen immune responses. Depletion of Tregs using anti-IL-2 receptor alpha chain antibody (anti-CD25 antibody) [19] potentiated vaccine induced immunity to tumors [19], [20], [21], [22]. Therapeutic immunization with a DC-based vaccine against HIV-1 induced a modest increase of Treg frequency and a significant increase in HIV-1-specific, Treg suppressive function. Thus, the Tregs suppressed the vaccine-induced anti-HIV-1-specific polyfunctional response [23]. In another system, depletion of Tregs increased the potency of plasmid DNA human-papillomavirus (HPV) vaccinations to control HPV-associated lesions [24].

NKT cells and Tregs regulate each other [25]. The same is true for NK/Treg interactions, which are also bidirectional [26], [27]. Since NK and NKT cells are the early responders to plasmid DNA vaccination at the site of inoculation [28] we aimed to determine if the response of these innate cell types is suppressed by Tregs during plasmid DNA vaccination. We therefore sequentially depleted these cells and measured the effect on plasmid DNA-elicited antigen-specific CD8^+^ T cell immune responses. We demonstrate that blocking the TGFβ-mediated suppression of NK cell activity by Tregs increases the CD8^+^ T cell immune response. These findings suggest a potential strategy to increase the potency of plasmid DNA vaccinations.

## Materials and Methods

### Ethics Statement

All animals were housed and maintained in accordance with the Guide for the Care and Use of Laboratory Animals [29], and all studies and procedures were reviewed and approved by the Institutional Animal Care and Use Committee (IACUC) of BIDMC (Chair Dr. Lisa Cavacini). BIDMC follows NIH guidelines for animal handling and has Animal Welfare Assurance A3153-01 on file with the Office for Protection of Research Risks. This institution maintains full accreditation from Association for Assessment and Accreditation of Laboratory Animal Care.

### Mice

Six- to eight-week-old female C57BL/6, NKT KO mice (on BALB/c background), BALB/c, and *beige* mice (C57BL/6-Lyst^bg^) [30], [31], [32] were purchased from The Jackson Laboratory. J18 KO mice (iNKT KO), which lack the V14-J18 NKT cells (lacking all invariant NKT (iNKT) cells), and NKT KO mice were both on a C57BL/6 background, and a gift from Dr. Mark E. Exley (Beth Israel Deaconess Medical Center (BIDMC), Harvard Medical School, Boston, MA). Both types of NKT KO mice are CD1d KO mice and lack all CD1d-restricted NKT cells [33].

### Vectors and Immunization

The plasmid DNA-Luciferase (DNA-Luc) construct with the AL11-epitope was prepared as previously described [34]. This vector contains the GL4.10 luciferase gene (Promega, Madison, WI) and the immunodominant H-2D^b^-restricted SIV-Gag AL11 epitope flanked by triple-alanine spacers. The complete CXCR4-tropic HIV-1 HXB2 Env IIIB (GenBank accession no. K03455) was cloned into the VRC vector (DNA-gp160) as previously described [35]. Plasmid DNA was prepared using an Endotoxin-free Qiagen Giga-prep kit (Qiagen, Valencia, CA). For immunizations, 50 µg of plasmid DNA in 100 µl of sterile saline was divided between quadriceps muscles by intramuscular (i.m.) inoculation. For sub-optimal DNA vaccinations, 20 µg of plasmid DNA was injected as described above. Endotoxin concentrations were determined with the E-Toxate kit (Sigma-Aldrich, St. Louis, MO), and were below 0.1 unit/µg in all plasmid DNA preparations used in these studies.

### Immunological assays

H-2D^b^/AL11 and H-2D^d^/p18 tetramers, which contain the SIV gag (AL11 peptide: AAVKNWMTQTL) or HIV gp160 (p18 peptide: RGPGRAFVTI) respectively, were prepared and used to identify the epitope-specific CD8^+^ T cells as previously described [35], [36]. Peripheral blood was collected and lysed with BD Pharm Lyse™ buffer (Becton-Dickinson, BD Biosciences, Mountain View, CA). Samples were analyzed by two-color flow cytometry on a FACSCalibur system (BD Biosciences). Gated CD8^+^ T lymphocytes were examined for staining with D^b^/AL11 or D^d^/p18 tetramer. CD8^+^ T lymphocytes from control mice immunized with untagged plasmid DNA-Luc exhibited <0.1% tetramer staining.

### Cell depletion and cytokine inactivation

For Treg cell inactivation experiments, C57BL/6 mice were injected by the intraperitoneal (i.p.) route with 0.5 mg of anti-CD25 antibody (clone 7D4) per infusion or, for the controls, with a corresponding isotype-matched non-specific rat IgM serum. Both antibody preparations were from BioXCell (West Lebanon, NH), and were injected 3 days prior to plasmid DNA inoculation and on the day of immunization. The degree of Treg depletion was measured by CD4^+^CD25^+^ staining for flow cytometric assays on days 1, 7, 14, 21 and 28; these assays utilized dye-coupled monoclonal anti-CD4-phycoerythrin (PE) (clone L3T4; BD Bioscience) and anti-CD25-allophyocyanin (APC) (clone PC61 5.3; Invitrogen, Carlsbad, Ca) antibodies [37]. Additionally, the efficiency of Treg depletion was measured by monoclonal antibody staining of isolated splenocytes with anti-CD25 APC-conjugated (clone PC61), anti-CD4-peridinin chlorophyll protein (PerCP) (clone L3T4; BD Bioscience) and anti-FoxP3 PE-conjugated (clone FJK-16S) antibodies (both BD Biosciences). Intra-cellular staining for Foxp3 was performed according to the manufacturer's protocol (BD Biosciences). Stained CD4^+^CD25^high^FoxP3^high^ cells were analyzed using the FACSCalibur system (BD Biosciences). Data were analyzed with FlowJo software (TreeStar, San Carlos, CA).

NK cell depletion was performed with the anti-asialo GM1 antibody (ASGM1) (Wako Chemicals, Richmond, VA) using 50-µl infusions as previously described [38] 3 days prior to and on the day of vaccination. Non-immune rabbit IgG (Sigma-Aldrich) was used as control. The anti-ASGM1 antibody depletion achieved greater than 50% depletion of NK cells over a 49 day period as confirmed by monoclonal anti-CD3 and anti-NK1.1 antibody (BD Bioscience) staining and flow cytometric analysis.

For TGFβ neutralization, C57BL/6 mice were injected by the i.p. route with 0.5 mg of anti-TGFβ (clone 1D11.16.8; BioXCell) or with a rat IgG1 isotype control (Sigma-Aldrich), 3 days before plasmid DNA inoculation and on the day of immunization. For IL-10 neutralization an anti-IL10 antibody (JESS-2A5, BioXCell) was used according to the same administration protocol. An isotype matched non-specific antibody was administered to control animals.

### Array for IL-10 gene expression

To analyze gene expression for Interleukin-10 (IL-10), the Oligo GEArray Mouse Microarray (SA Biosciences, Frederick, MD, cat# OMM-012,) was used on snap-frozen vaccinated or unvaccinated quadriceps muscles 14 days after DNA vaccination according to the manufacturer's protocol.

### Data analysis

Differences between groups were analyzed using the Mann-Whitney test. A *p* value of <0.05 was considered significant. Statistical calculations were performed using the GraphPad Prism program (version 4.03). Error bars represent the standard errors of the means (SEM).

## Results

### Tregs dampen plasmid DNA antigen-specific CD8^+^ T cell immune responses

CD4^+^CD25^+^ Tregs not only restrict self-antigen-specific immune responses, but also dampen immunity against foreign-antigens. Therefore, depletion or inactivation of Tregs might help increase antigen-specific T cell immune responses elicited by a plasmid DNA vaccine. One possible method of depleting or inactivating Tregs involves treatment with a monoclonal anti-CD25 antibody, the effects of which include loss of CD4^+^CD25^+^ cells *in vivo* as monitored by flow cytometry [37]. Previous studies have shown that this treatment results in the reliable enhancement of antigen-specific CD8^+^ T cell immune responses elicited by a plasmid DNA vaccine [24].

**Figure 1:**
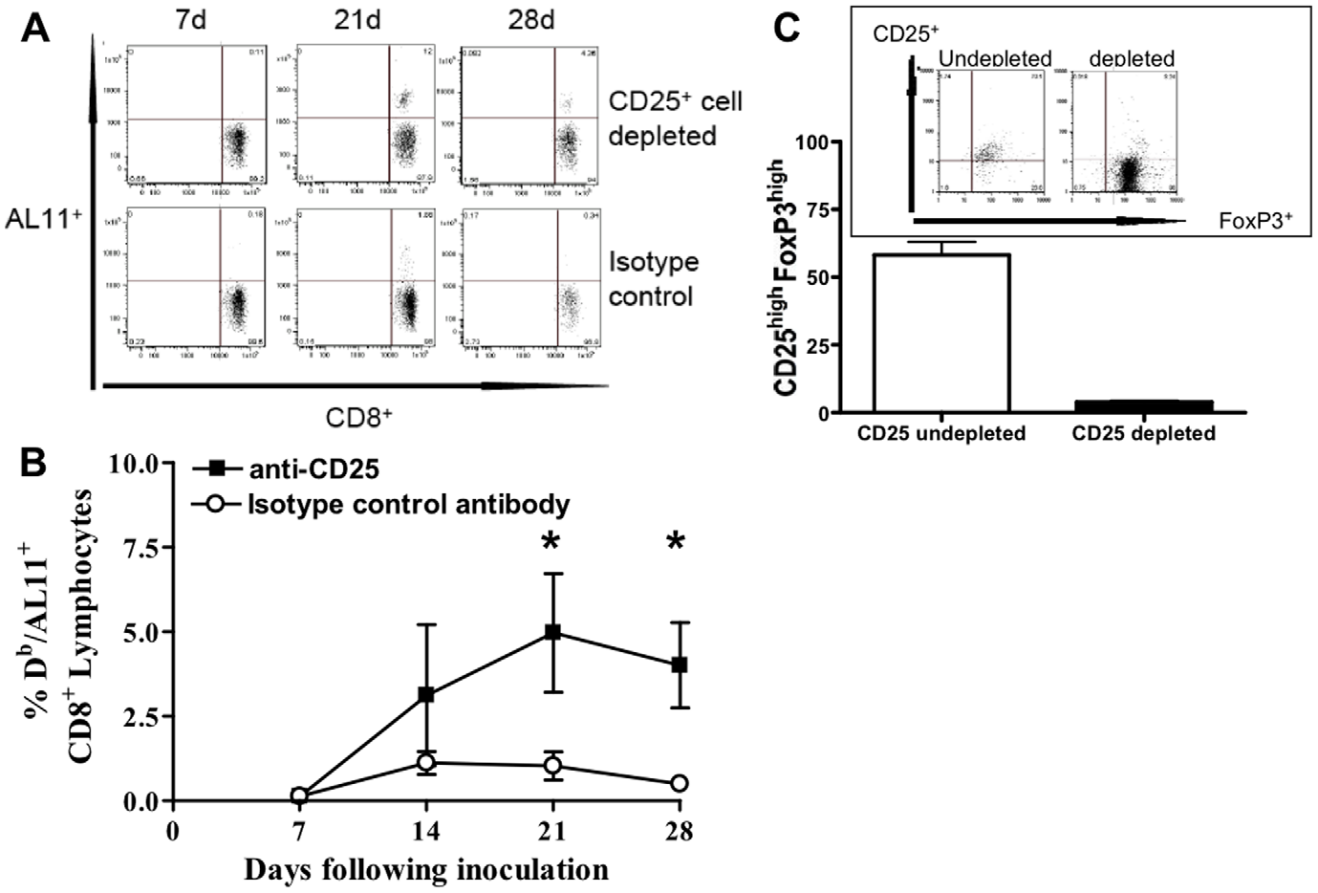
Damping of the antigen-specific CD8^+^ T cell immune response is mediated by a CD25^+^ T cell. (A) Representative gating of AL11 tetramer stains of CD25^+^ depleted or undepleted mice (isotype control) after plasmid DNA vaccination. (B) SIV-Gag AL11 epitope-specific CD8^+^ T cell responses in vaccinated anti-CD25- and isotype-matched antibody-treated mice. (C) Percentage of CD4^+^CD25^high^ FoxP3^high^ cells at day 7 with inset showing representative gating of splenocytes of mice depleted or undepeted of CD25^+^ cells. Epitope-specific CD8^+^ T cell responses were measured by D^b^/AL11 tetramer staining of CD8^+^ T cells in 5 C57BL/b6 mice per group at the indicated times following plasmid DNA vaccine construct inoculation. CD25^+^ cells were inactivated by administration of anti-CD25 antibody 3 days prior to and on the day of vaccination. Line graphs represent mean values, and error bars represent SEM. Statistically significant differences at specific times were determined by Mann-Whitney test. Significant differences are indicated by asterisks (p<0.05).

To determine whether CD25^+^ cell depletion can potentiate an SIV-specific CD8^+^ T cell immune response, we immunized mice with a plasmid DNA-Luc construct encoding the H-2D^b^-restricted cytotoxic T lymphocyte (CTL) epitope of SIV gag (AL11) and measured the subsequent immune response by tetramer staining using flow cytometry. Following anti-CD25 antibody treatment, we found AL11-specific immune responses increased an average of 2- to 4-fold (Fig. 1A/B). In our experiments monoclonal anti-CD25 antibody infusions led to a 3 week-long 95% reduction in detectable CD4^+^CD25^+^ cells in the peripheral blood. Treg depletion can also be measured through the reduction of the Treg transcription factor FoxP3 and CD25 expression. Isotype antibody treated, undepleted mice splenocytes showed at day 7, 58.2±4.8% CD4^+^CD25^high^ FoxP3^high^ cells, which were more than 90% reduced after anti-CD25 antibody infusion (3.8±0.6%, p = 0.008) (Fig. 1C).

### Tregs have no major influence on NKT cell mediated immune responses

**Figure 2:**
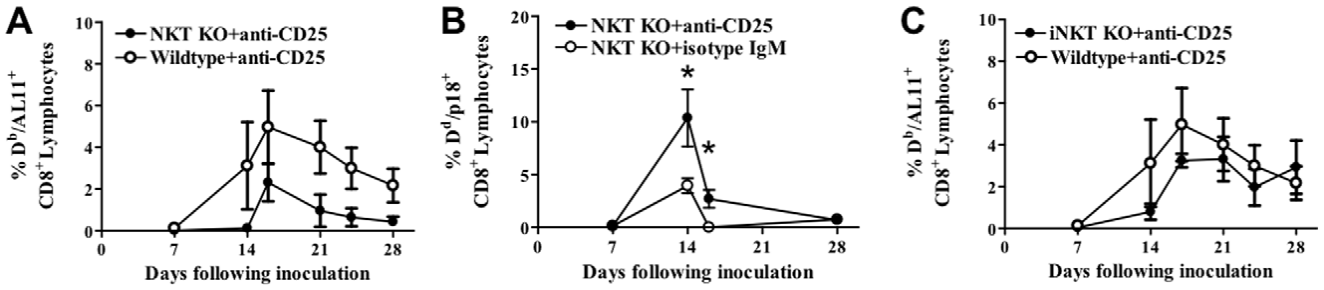
Damping of the antigen-specific CD8^+^ T cell immune response after anti-CD25 antibody treatment in NKT and iNKT KO mice. Effect of Treg inactivation on the plasmid DNA antigen-specific CD8^+^ T cell immune response in (A) wildtype and NKT KO mice (C57BL/6 mice, both Treg inactivated); in (B) NKT KO mice (BA LB/c mice) treated with an anti-CD25 or an isotype-matched antibody; in (*C*) wildtype and iNKT KO mice (C57BL/6 mice, both Treg inactivated). For anti-CD25 antibody treatment, mice were injected via the i.p. route with an anti-CD25 antibody 3 days prior to and on the day of vaccination. Epitope-specific CD8^+^ T cell responses were measured in 5 mice per group by D^b^/AL11 (A, C) or D^d^/p18 (B) tetramer staining of CD8^+^ T cells at the indicated times following plasmid DNA vaccine inoculation. Line graphs represent mean values, and error bars represent SEM. Statistically significant differences at specific times were determined by Mann-Whitney test. Significant differences are indicated by asterisks (p<0.05).

We previously found that the antigen-specific CD8^+^ T cell immune response to plasmid DNA vaccine is dependent on early innate immune responses [39]. Early innate responders at the site of inoculation include NKT and NK cells [28]. Cytokine release by NKT cells was found to be important to initiate a potent antibody and T cell immune response to plasmid DNA vaccines [28], [40], [41]. Tregs may suppress NKT immune action at the site of injection. To investigate this possibility, we infused anti-CD25 antibodies 3 days before vaccination and on the day of plasmid DNA vaccination. We hypothesized that, if Treg suppression of vaccine-elicited CD8^+^ T-cell responses was due primarily to inhibition of NKT cell function, anti-CD25 antibody treatment would not enhance CD8^+^ T cell immune responses in NKT KO mice. We investigated the effect of Tregs on NKT cells by using three different strains of mice and two different antigens. Our first approach at measuring the effect of Tregs on NKT cells used a system in which Treg-depleted NKT KO mice are compared to Treg-depleted wildtype mice (Fig. 2A). If Tregs suppress the NKT cell-mediated augmentation of the CD8^+^ T cell response, we would expect to observe a significant difference in the magnitude of the CD8^+^ T cell response between the NKT KO and wildtype mice. We found no significant difference between Treg-depleted wildtype mice or NKT KO mice (d21: *p* = 0.14, d24: *p* = 0.11, d28: *p* = 0.14; 5 mice per group) using C57BL/6 mice and the AL11 antigen; this finding eliminated the possibility that NKT cells might be the primary targets of Tregs (Fig. 2A). Another approach to measuring the effect of Tregs is by depleting them in NKT KO mice. If Tregs suppress NKT cells, the Treg-depleted mice, compared to undepleted NKT KO mice, should show no enhancement in the CD8^+^ T cell immune response. We found that BALB/c mice vaccinated with the gp160 env antigen and treated with anti-CD25 antibody infusions had a significant increase in the CD8^+^ T cell immune response compared to the control group. This confirmed our above findings that the suppression of the plasmid DNA-induced CD8^+^ T cell response by Tregs is not due to suppression of NKT cells (Fig. 2B). We then used a third model of iNKT KO mice to test the effects of Tregs on NKT cells. These mice lack iNKT, a subset of NKT cells involved in most immune effects attributed to NKT cells. We found that depletion of Tregs had no effect on CD8^+^ T cell immune responses, a result that ruled out iNKT cells as key players in Treg-mediated plasmid DNA CD8^+^ T cell immune suppression (Fig. 2C). While our experiment show that Treg have an impact on CD8^+^ T-cell responses in NKT KO mice, it does not rule out a partial inhibition NKT cells by Treg during plasmid DNA vaccination.

### Tregs suppress NK cells, resulting in a damping of plasmid DNA-specific CD8^+^ T cell immune responses

**Figure 3:**
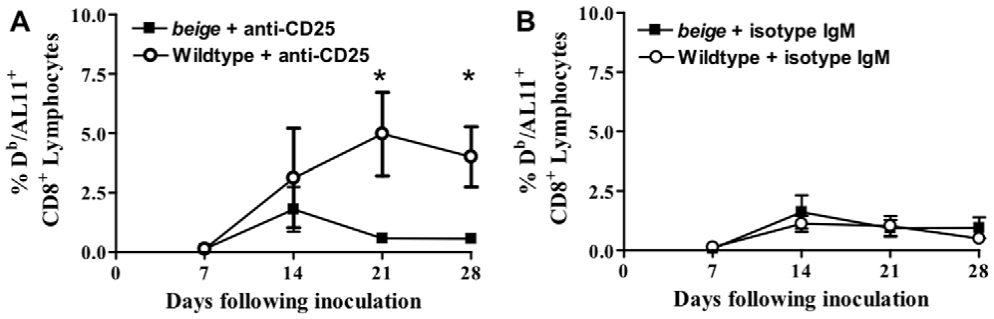
Damping of antigen-specific CD8^+^ T cell immune response after anti-CD25 antibody treatment in NK-function KO mice. (A) Plasmid DNA antigen-specific T cell immune responses in vaccinated wildtype and *beige* mice (C57BL/6 mice), all treated with an anti-CD25 antibody. (B) Plasmid DNA antigen-specific CD8^+^ T cell immune responses in wildtype and *beige* mice, both groups treated by isotype-matched control antibodies (isotype IgM). For anti-CD25 antibody treatment, mice were injected via the i.p. route with an anti-CD25 antibody 3 days prior to and on the same day of vaccination. Epitope-specific CD8^+^ T cell responses were measured by D^b^/AL11 staining of CD8^+^ T cells in 5 mice per group at the indicated times following plasmid DNA vaccine construct inoculation. Line graphs represent mean values, and error bars represent SEM. Statistically significant differences at specific times were determined by Mann-Whitney test. Significant differences are indicated by asterisks (p<0.05).

NK cells are potential targets of Treg suppression. This phenomenon was demonstrated in cancer models in which Tregs were found to block NK cell function. When Tregs were depleted by anti-CD25 antibody treatment, the anti-cancer activity of NK cells was restored [10], [42]. To test the influence of Tregs on NK cells following plasmid DNA vaccination, we depleted Tregs from NK cell function-deficient *beige* mice and wildtype mice. Following Treg depletion, we found that NK cell function-deficient mice were not able to increase AL11-specific CD8^+^ T cell immune responses in contrast to the wildtype mice (Fig. 3A). This finding demonstrated that NK cells might be suppressed by Tregs during plasmid DNA vaccination and could explain the potentiating effect of Treg depletion found for plasmid DNA vaccination, a effect not found when control antibodies were used (Fig. 3B).

**Figure 4:**
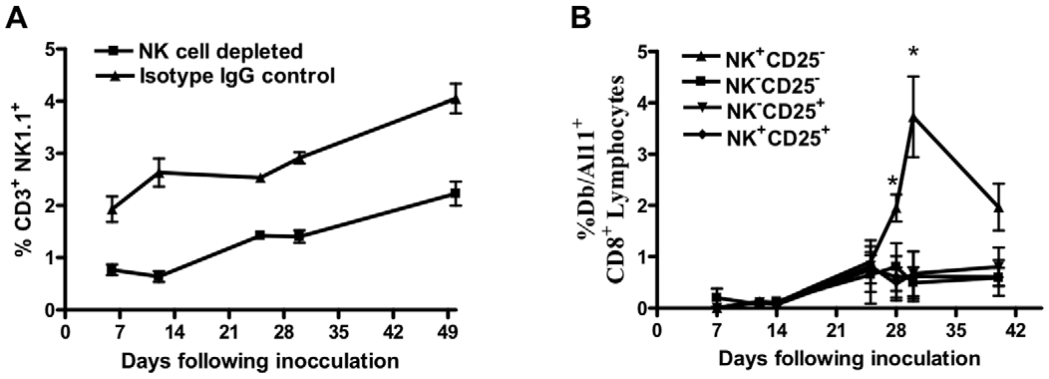
Antigen-specific CD8^+^ T cell immune response is increased in Treg-depleted mice where NK cell compartment is intact. (*A*) Flow cytometric analysis of NK cells (CD3^−^, NK1.1^+^) after plasmid DNA vaccine inoculation and anti-ASGM1 antibody NK cell depletion. (*B*) SIV-Gag AL11 epitope-specific CD8^+^ T cell responses in four groups of 5 C57BL/6 mice each to characterize the NK/Treg suppression: NK undepleted and CD25 depleted (NK^+^CD25^−^), NK and CD25 depleted (NK^−^CD25^−^), NK depleted and CD25 undepleted (NK^−^CD25^+^) and NK undepleted and CD25 undepleted (NK^+^CD25^+^). Anti-CD25 antibody and anti-ASGM1 antibody treatment was done 3 days prior and on the day of plasmid DNA vaccination. Line graphs represent mean values, and error bars represent SEM. Statistically significant differences at specific times were determined by Mann-Whitney test. Significant differences are indicated by asterisks (p<0.05).

To determine if this effect is NK cell-dependent, and not an effect of the idiosyncrasy of the NK-function deficient mice, we depleted NK cells with anti-ASGM1 antibodies. We found that NK cells were depleted at least 50% over a 49 day period when anti-ASGM1 antibody treated, NK cell-depleted wildtype mice were compared to isotype antibody-treated control mice (Fig. 4A). We immunized wildtype and anti-ASGM1 antibody-treated mice with suboptimal amounts of plasmid DNA to more closely simulate the real-time situation during human vaccination and to increase Treg effects compared to controls. We used four groups of mice to characterize the suppression of NK cells by Tregs: NK-undepleted, CD25-depleted (NK^+^CD25^−^); NK- and CD25-depleted (NK^−^CD25^−^); NK-depleted, CD25-undepleted (NK^−^CD25^+^) and NK-and CD25-undepleted (NK^+^CD25^+^). We found that CD8^+^ T cell immune responses were delayed. We also found that plasmid DNA vaccine-elicited CD8^+^ T cell immune responses increased up to 7-fold when Tregs were depleted but the NK cell compartment was kept intact (NK^+^CD25^−^), while simultaneous depletion of Tregs abolished this effect (Fig. 4B). This further confirmed our above findings in the NK-function deficient mice that NK cell function is suppressed by Tregs.

### Tregs suppression is IL-10-independent

**Figure 5:**
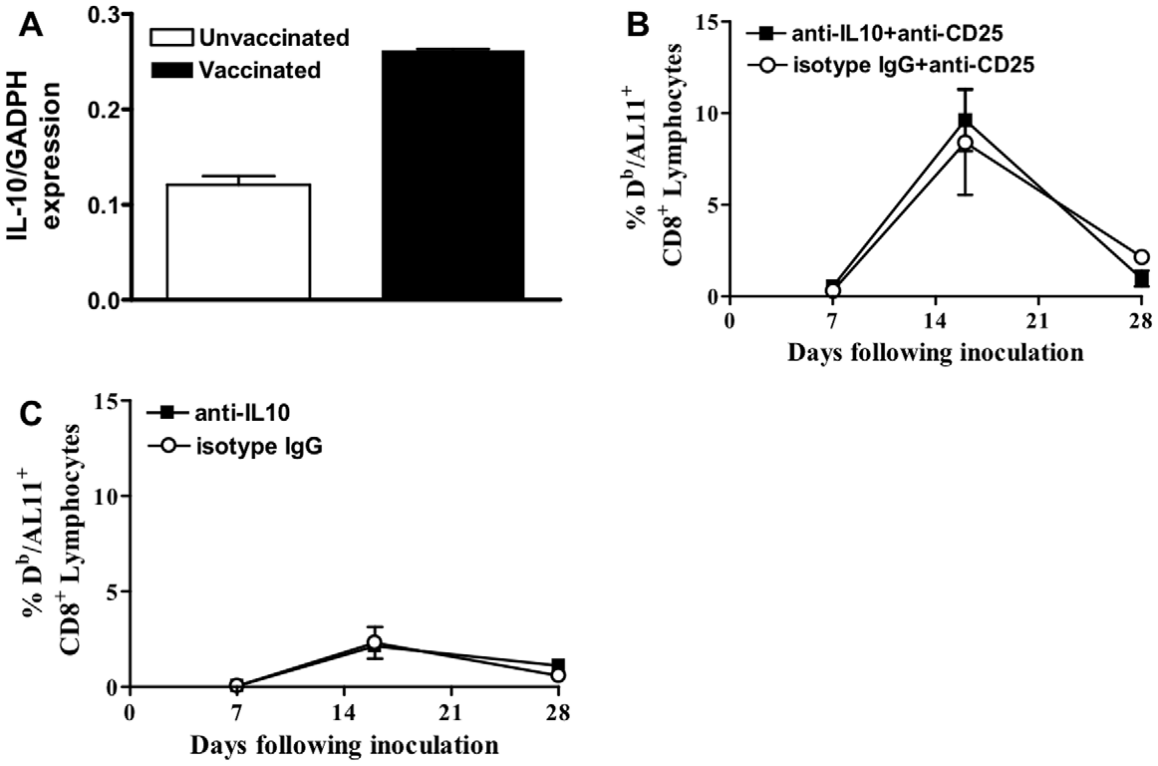
The antigen-specific CD8^+^ T cell immune response is independent of IL-10 neutralization. (A) IL-10 expression in relation to expression of the house-keeping gene GADPH in plasmid DNA vaccinated and unvaccinated mice (C57BL/6 mice). (B) T cell immune responses in vaccinated IL-10-neutralized or isotype matched antibody-treated (isotype IgG) wildtype C57BL/6 mice treated with an anti-CD25 antibody. (C) T cell immune responses in vaccinated IL-10-neutralized or isotype matched antibody-treated (isotype IgG) wildtype C57BL/6 mice not treated with an anti-CD25 antibody. Epitope-specific CD8^+^ T cell responses were measured in 5 mice per group by D^b^/AL11 tetramer staining of CD8^+^ T cells at the indicated times following plasmid DNA vaccine inoculation. Line graphs represent mean values, and error bars represent SEM. Statistically significant differences at specific times were determined by Mann-Whitney test. Significant differences are indicated by asterisks (p<0.05).

Immune suppression *in vivo* is mediated by Tregs' release of cytokine IL-10 and/or TGFβ [13], [18]. To investigate if there is a change in IL-10 production in vaccinated mice muscle cells which might be responsible for the change in plasmid DNA vaccine elicited CD8^+^ T cell immune response, we measured the gene-expression of IL-10. We found that IL-10 expression was increased up to 3-fold in muscles of vaccinated animals compared to unvaccinated animals. IL-10 may be released by Tregs and could potentially be responsible for the blunting of the CD8^+^ T cell plasmid DNA immune response (Fig. 5A). Therefore, we investigated the impact of IL-10 depletion on vaccine-elicited CD8^+^ T cell immune response in the presence or absence of Treg. If the release of IL-10 by Tregs during plasmid DNA vaccination has an effect on the vaccine-elicited CD8^+^ T cell immune response, we would expect a stronger immune response in wildtype mice, but not CD25-depleted mice treated with the anti-IL10 antibodies. We found that administration of IL-10-neutralizing antibodies during the early phase of immunization with or without Treg depletion had no effect on the CD8^+^ T cell immune response (Fig. 5B/C); we, therefore, concluded that this cytokine is not responsible for the Treg-mediated suppression of vaccine-elicited CD8^+^ T-cell responses.

### Tregs suppression is TGFβ-dependent

**Figure 6:**
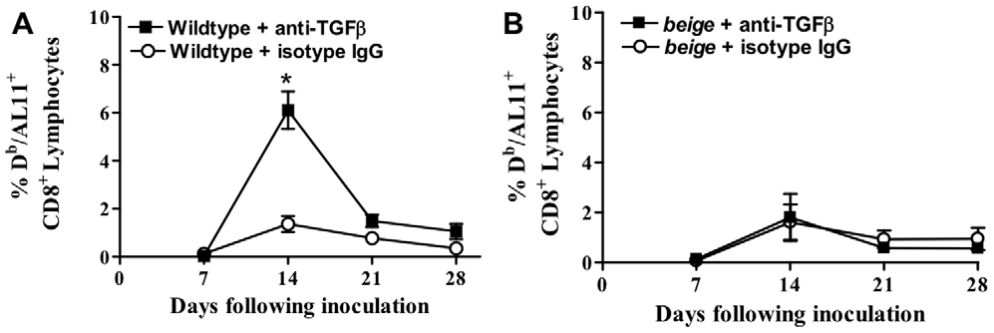
The antigen-specific CD8^+^ T cell immune response is dependent on TGFβ neutralization. (*A*) Plasmid DNA antigen-specific CD8^+^ T cell immune responses after TGFβ-neutralization by specific antibody (anti-TGFβ) in wildtype C57BL/6 mice; control group treated with an isotype matched antibody (isotype IgG) is also shown. (*B*) Plasmid DNA antigen-specific CD8^+^ T cell immune responses after TGFβ-neutralization by specific antibody (anti-TGFβ) in *beige* mice; control group treated with an isotype-matched control antibody (isotype IgG) is also shown. TGFβ was neutralized via i.p. administration of anti-TGFβ neutralizing antibody 3 days prior to and on the same day of vaccination. Epitope-specific CD8^+^ T cell responses were measured in 5 mice per group by D^b^/AL11 tetramer staining of CD8^+^ T cells at the indicated times following plasmid DNA vaccine inoculation. Line graphs represent mean values, and error bars represent SEM. Statistically significant differences at specific times were determined by Mann-Whitney test. Significant differences are indicated by asterisks (p<0.05).

To determine if the suppressive effect of Tregs is mediated by TGFβ, we neutralized this cytokine with infusions of TGFβ neutralizing antibodies. We found that neutralization of TGFβ increased the vaccine-elicited CD8^+^ T cell immune response compared to animals treated with an isotype-matched non-specific control antibody (Fig. 6A). To determine whether TGFβ exerts its effects on vaccine-elicited CD8^+^ T cell responses through NK cells, we treated NK function-deficient *beige* mice with anti-TGFβ antibodies. TGFβ neutralizing antibodies had no effect on vaccine antigen-specific CD8^+^ T cell immune responses in the NK function-deficient mice (Fig. 6B). This demonstrated that TGFβ suppressed vaccine-elicited CD8^+^ T cell responses by inhibiting NK cell function. To demonstrate that Tregs are the source of the suppressive TGFβ, we depleted mice of CD25^+^ cells and neutralized TGFβ using both antibodies at the same time. In another control experiment we compared the SIV-Gag-specific CD8^+^ T cell response in mice treated with both antibodies to control mice treated with the anti-CD25 antibody and the TGFβ isotype control antibody. We found that both groups had similar plasmid DNA antigen-specific CD8^+^ T cell immune responses (d7: *p* = 0.26, d10: *p* = 0.1, d14: *p* = 0.73; 5 mice per group) suggesting that the TGFβ effect is indeed produced by the Tregs.

## Discussion

Emerging data suggest that Tregs are a unique population of T cells that maintain immune tolerance and provide negative feedback for effector T cell immune responses [19], [20], [21], [22]. Augmentation of the antigen-specific CD8^+^ T cell immune response was reported for a plasmid DNA vaccine following depletion of Tregs [24]. This observation provided the rationale for investigating the cellular targets and effector mechanisms involved in Treg-mediated suppression of plasmid DNA vaccine efficacy.

Forty-eight hours following plasmid DNA vaccination, NK and NKT cells migrate to the vaccine injection site [28], and an early innate immune response is initiated, leading to an increase in efficacy of plasmid DNA vaccination [39]. However, only NKT cells seem to be involved in potentiating these CD8^+^ T cell immune responses [40], [41]. NK cells although being early at the site of inoculation seem to have no effect on CD8^+^ T cell plasmid DNA immune responses [41], [43]. We, therefore, hypothesized that Tregs may suppress NK cell-mediated immune activation.

NKT cells and Tregs regulate each other through the release of cytokines [25]. Our current data indicate that suppression of NKT cells by Tregs is not the major cause for the decrease of plasmid DNA vaccine efficacy. We found that when Tregs are depleted in NKT KO mice, the plasmid DNA-elicited CD8^+^ T-cell immune response is increased, suggesting other cell types are being suppressed by Tregs. Nevertheless, there was a trend towards diminished CD8^+^ T cell immune responses in NKT KO mice as compared to wildtype mice after Treg depletion. Additionally, after Treg depletion, NKT KO mice showed a more rapidly decline of CD8^+^ T cell immune response than was observed for wildtype mice. This diminished CD8^+^ T cell immune response might be the result of the lack of T_H_1 immune support by MCP-1, a major chemokine early released by NKT cells after plasmid DNA inoculation and found to be important for the magnitude of the vaccine-elicited immune response [41].

Earlier studies suggest a role for Tregs in suppressing NK cell effector function *in vitro* [6]. Tregs have also been shown to suppress NK cells *in vivo* by directly inhibiting NKG2D-mediated NK cell cytotoxicity [10]. TGFβ, released by Tregs, is known to confer this suppressive effect on NK cell-mediated cytotoxicity. This was demonstrated when infusion of anti-TGFβ monoclonal antibodies restored the NK cell-mediated cytotoxicity towards RMA-Rae-1 tumor cells. However, neutralization of IL-10, also released by the Tregs, did not appear to modify NK cell function [10]. The pathological relevance of the Treg–NK cell interaction has been evaluated in several tumor models and in patients with cancer. Consequently, inhibition of Tregs through pharmacological interventions is considered during NK-cell-based immunotherapy of cancer [44]. Our data expand on these *in vivo* findings and demonstrate that NK cell functions are suppressed by Tregs through the release of T_H_3 cytokine TGFβ during plasmid DNA vaccination. This inhibition of NK cell function leads to a blunting of the CD8^+^ T cell immune response.

Recent studies have identified important roles for Tregs and NK cells in the pathogenesis of biliary atresia in mice. Early after perinatal rhesus rotavirus infection, the liver is devoid of Tregs. Not only does this result in an absence of the Tregs' suppressive effect on DC-dependent activation of naive NK cells, but it also prevents Tregs from suppressing hepatic NK cell expansion. [45]. Thus, the post-natal absence of Tregs may be a key factor that allows hepatic DCs and NK cells act unopposed in initiating neonatal bile duct injury. Therefore, our findings might also be the result of DC activation after Treg depletion, resulting in a stronger NK cell immune response, which in turn might increase the plasmid DNA vaccine elicited CD8^+^ T cell immune response.

CpG motifs augment DNA vaccine potency by induction of MCP-1 secretion [28]. Toll-like receptor (TLR) ligands are notable for their ability to induce antigen presenting cells (APC) maturation, which in turn facilitates optimal T cell mediated immune responses. TLR ligands, such as the CpG motifs, can also modulate immune responses by blocking the suppressive effects of Tregs through direct MyD88-dependent costimulation of effector CD4^+^ T cells, leading to enhanced IL-2 production [46]. These augmented CD4^+^ T cell immune responses might then increase plasmid DNA vaccine potency; a phenomenon described earlier [43].

In summary, our data indicate a crucial function for Tregs in blocking plasmid DNA-elicited immune responses. Additionally, we demonstrate that these responses can be potentiated by Treg depletion or TGFβ neutralization, greatly enhancing the efficacy of the plasmid DNA vaccine modality. Potent plasmid DNA vaccine vectors are necessary in vaccine regiments because they elicit no immune responses against their backbone, as can be the case with viral vectors. Unwanted immune responses, therefore, are minimized. As a result of our findings, strategies may be developed that improve the immune response to plasmid DNA vaccination. Such strategies might include a step in which Tregs are depleted at the site of inoculation by chemicals or depleting antibodies.
